# Renal hemangiopericytoma secondary to refractory hypertension in a child: A case report

**DOI:** 10.3892/ol.2014.2577

**Published:** 2014-09-29

**Authors:** QINGFENG HU, ZUJUN FANG, ZHONGWEN ZHOU, JIE ZHENG

**Affiliations:** 1 Department of Urology, Huashan Hospital, Fudan University, Shanghai 200040, P.R. China; 2 Department of Pathology, Huashan Hospital, Fudan University, Shanghai 200040, P.R. China

**Keywords:** hemangiopericytoma, kidney neoplasm, hypertension, nephrectomy

## Abstract

Hemangiopericytoma is a rare perivascular tumor that often involves the extremities, pelvis, head and neck, and meninges, but rarely occurs in the kidney. The differentiation from renal cancer prior to surgery is extremely challenging; therefore, almost all cases of renal hemangiopericytoma are diagnosed by pathological examination. The majority of cases are identified in patients between the ages of 20 and 50 years of age, and a considerable proportion of patients exhibit hypertension, hypoglycaemia or additional paraneoplastic syndromes. The current study reports a rare case of renal hemangiopericytoma with drug refractory hypertension in a 14-year-old female. Following the complete resection of the tumor, the patient’s blood pressure returned to normal. No evidence of recurrence or metastasis was observed during a follow-up of 12 months following surgery. The present case indicated that surgery provides satisfactory outcomes and appears to be the most effective modality of treatment for renal hemangiopericytoma. Furthermore, this case also demonstrated that secondary hypertension may also recover following tumor excision.

## Introduction

Hemangiopericytoma is a rare perivascular tumor in which uncontrolled proliferation of pericytes occurs, which often involves the extremities, pelvis, head and neck, and meninges, but rarely occurs in the kidney; renal hemangiopericytoma has been reported in ≤50 cases since the initial case was reported by Black and Heinemann in 1955 (1). The majority of cases have been identified in patients between 20 and 50 years of age, and a considerable proportion of patients present with hypertension, hypoglycaemia or additional paraneoplastic syndromes (2). Due to the rarity of the tumor, the exact diagnosis, effective treatment and prognosis of the tumor remain unclear. The current study reports a rare case of hemangiopericytoma with drug refractory hypertension in a 14-year-old female. Written informed consent was obtained from the patient’s family.

## Case report

**Figure 1:**
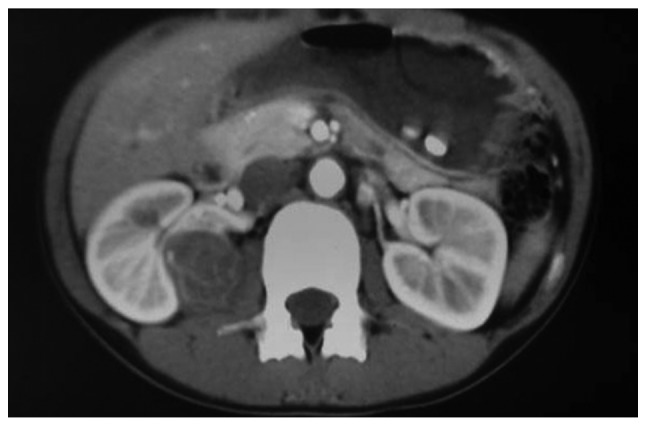
Computed tomography for the right renal hemangiopericytoma.

A 14-year-old female was admitted to Huashan Hospital, Fudan University (Shanghai, China) with intermittent dizziness and vomiting for the previous three months. The patient exhibited hypertension, with a blood pressure of 200/140 mmHg. However, the blood pressure continued to fluctuate above 150/100 mmHg following treatment with losartan, nifedipine and aldactone for over six weeks. The levels of renin (1.5 μg/l/h; normal range, 1.0–2.5 μg/l/h) and angiotensin (22 ng/l; normal range, 10–30 ng/l) were not increased, and the serum creatinine (56 μmol/l; normal range, 44–133 μmol/l), sodium (139 mmol/l; normal range, 135–147 mmol/l) and potassium (4.1 mmol/l; normal range, 3.5–5.5 mmol/l) levels, together with C-reactive protein (0.74 mg/l; normal range, 0–3.25 mg/l) levels and erythrocyte sedimentation rate (13 mm/h; normal range, 0–20 mm/h), were also within normal ranges. Abdominal ultrasonography revealed an isoechoic solid lesion of 3.5 cm in diameter in the center of the right kidney, and computed tomography also showed a mass with abnormal density, particularly in the arterial and venous phase (Fig. 1). No tumor infiltration was identified in the renal collecting system, vessels and perirenal tissue, as well as retroperitoneal lymph nodes.

**Figure 3:**
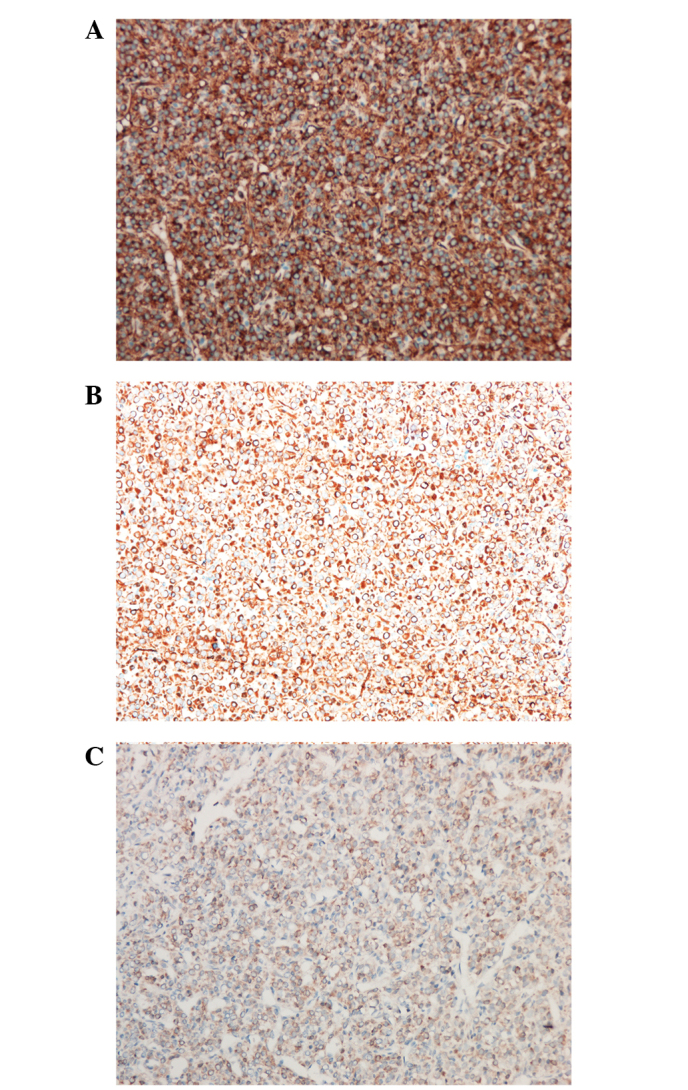
Immunohistochemical staining was positive for (A) CD34, (B) vimentin and (C) Bcl-2 (magnification, 10×20).

Partial nephrectomy with an open, lumbotomic approach was implemented for this renal lesion with unknown characteristics. An exophytic and clear, circumscribed tumor was excised completely. The specimen was 3.5 cm in diameter, and a homogeneous texture without necrosis or cystic separation was visible on gross examination. On microscopic examination, monotonous proliferation with no significant variability and pericytes around the endothelial vascular channels were the characteristic features, which indicated renal hemangiopericytoma (Fig, 2). The positive results of vimentin, Bcl-2 and CD34 by immunohistochemical staining also supported this diagnosis (Fig. 3).

The duration of hospitalization was six days and no perioperative complications were observed during that time. After a follow-up of 12 months, the patient remains well with no evidence of recurrence or metastasis; the blood pressure has returned to within the normal range (115/70 mmHg) and no antihypertensive drugs are in use.

## Discussion

Hemangiopericytoma is a rare vascular tumor of the soft tissue originating from pericytes and was initially described by Zimmermann in 1923 (3). It has been demonstrated to be a monotonous cellular proliferation with no significant variability, and exhibits pericytes around endothelial vascular channels with collagenization. Immunohistochemical analysis provides substantial information as positive reactions for antibodies including CD34 and vimentin are characteristic of cells of mesenchymal origin and thus, they are widely used to identify neoplastic progenitor cells surrounding vascular spaces (4). A combination of histological and immunohistochemical patterns may provide an exact diagnosis.

Previous studies have found that the mean age of patients at the time of diagnosis and surgery is 40 years (2). In the pressent study, the patient was 14 years old and thus, at present this is the youngest patient reported. The age distribution indicates that renal hemangiopericytoma often affects younger patients.

It is difficult to differentiate renal hemangiopericytoma from RCC using current imaging technology (2). By contrast to other tumors, numerous patients present with paraneoplastic syndromes, including hypertension, hypoglycemia, electrolyte disorders and cachexia (2). Hypertension is the most common symptom, however, its association with renal hemangiopericytoma remains unclear. Robertson *et al* (5) hypothesized that it is a result of the renin produced by the tumor (5). However, in the present case the patients did not present with a high level of renin. Patients with juxtaglomerular cell tumors often present hypertension, which is due to the secretion of renin. Juxtaglomerular cell tumors, also termed reninoma, are tumors of the renal juxtaglomerular cell apparatus, which causes hypertension and hypokalemia due to the hypersecretion of renin (6). The diagnosis of hemangiopericytoma is usually the result of excluding the possibility of other vascular and mesenchymal tumors, according to the histological pattern and the immunohistochemical results (2). Although this tumor may also present with hypertension, it is considered to have a different origin from that of hemangiopericytoma (2).

Although radiotherapy and other modalities may be performed (7), surgery is considered the most effective treatment, and has been performed in every case reported (2). Renal hemangiopericytoma usually grows insidiously without evident symptoms, and the majority patients receive radical nephrectomy (2). As aforementioned, this tumor is not easily differentiated from RCC, and thus all surgical procedures must comply with those of RCC. Partial nephrectomy is recommended, when feasible during surgery, as it may provide improved renal function and oncological outcomes, when compared with radical nephrectomy (8,9). Therefore, patients with renal hemangiopericytoma may experience a longer disease-free or progression-free survival following surgery and hypertension associated with the tumor may also recover (5,10,11).

In conclusion, renal hemangiopericytoma is a rare perivascular tumor and patients may present with hypertension or other paraneoplastic syndromes. The results of the present case indicated that surgery provides satisfactory outcomes for patients with renal hemangiopericytoma and it appears to be the most effective modality of treatment for the disease. Furthermore, this case demonstrated that secondary hypertension may also recover following surgery.

## Figures and Tables

**Figure 2:**
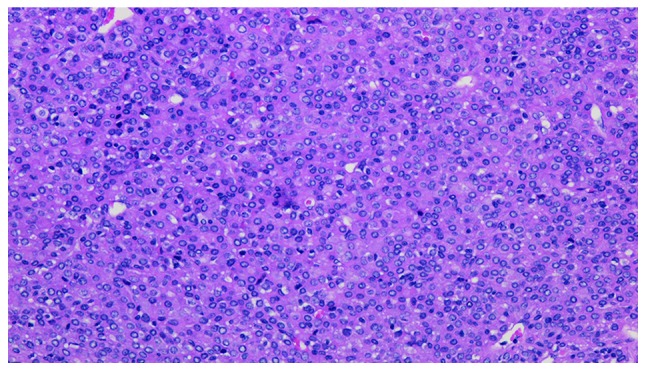
Microscopic examination in absence of immunohistochemical staining (stain, hematoxylin and eosin; magnification,10×20).
